# MILO/ENGOT-ov11: Binimetinib Versus Physician’s Choice Chemotherapy in Recurrent or Persistent Low-Grade Serous Carcinomas of the Ovary, Fallopian Tube, or Primary Peritoneum

**DOI:** 10.1200/JCO.20.01164

**Published:** 2020-08-21

**Authors:** Bradley J. Monk, Rachel N. Grisham, Susana Banerjee, Elsa Kalbacher, Mansoor Raza Mirza, Ignacio Romero, Peter Vuylsteke, Robert L. Coleman, Felix Hilpert, Amit M. Oza, Anneke Westermann, Martin K. Oehler, Sandro Pignata, Carol Aghajanian, Nicoletta Colombo, Esther Drill, David Cibula, Kathleen N. Moore, Janna Christy-Bittel, Josep M. del Campo, Regina Berger, Christian Marth, Jalid Sehouli, David M. O’Malley, Cristina Churruca, Adam P. Boyd, Gunnar Kristensen, Andrew Clamp, Isabelle Ray-Coquard, Ignace Vergote

**Affiliations:** ^1^ Arizona Oncology (US Oncology Network), University of Arizona College of Medicine, Creighton University School of Medicine, Phoenix, AZ; ^2^ Memorial Sloan Kettering Cancer Center, Weill Cornell Medical Center, New York, NY; ^3^ Royal Marsden National Health Service Foundation Trust and Institute of Cancer Research, London, United Kingdom; ^4^ Centre Hospitalier Régional et Universitaire de Besançon, CHRU de Besançon, Besançon, France; ^5^ Nordic Society of Gynaecological Oncology and Rigshospitalet, Copenhagen University Hospital, Copenhagen, Denmark; ^6^ Servicio de Oncologıa Medica, Fundacion Instituto Valenciano de Oncologıa, Valencia, Spain; ^7^ CHU Université catholique de Louvain Namur, Sainte-Elisabeth, Namur, Belgium; ^8^ University of Botswana, Gaborone, Botswana; ^9^ MD Anderson Cancer Center, Houston, TX; ^10^ Onkologisches Therapiezentrum am Krankenhaus Jerusalem, Hamburg, Germany; ^11^ Princess Margaret Cancer Centre, Toronto, Ontario, Canada; ^12^ Dutch Gynaecological Oncology Group, Amsterdam University Medical Centers, Amsterdam, the Netherlands; ^13^ Department of Gynaecological Oncology, Royal Adelaide Hospital, Adelaide, South Australia 5005, Australia; ^14^ Istituto Nazionale Tumori Fondazione Pascale IRCCS, Naples, Italy; ^15^ Dipartimento Medicina e Chirurgia, Università Milano-Bicocca, Programma Ginecologia Oncologica Istituto Europeo Oncologia, IRCCS, Milan, Italy; ^16^ First Faculty of Medicine, Charles University in Prague and General University Hospital, Prague, Czech Republic; ^17^ Stephenson Cancer Center at The University of Oklahoma Health Sciences Center, Oklahoma City, OK; ^18^ Pfizer, New York, NY; ^19^ Vall d'Hebron University Hospital, Barcelona, Spain; ^20^ University Clinic for Gynaecology and Obstetrics, Medical University of Innsbruck, Innsbruck 6020, Austria; ^21^ Department of Obstetrics and Gynecology, Medical University of Innsbruck, Austrian AGO, Innsbruck, Austria; ^22^ Center for Oncological Surgery, European Competence Center for Ovarian Cancer Campus Virchow Klinikum and Benjamin Franklin Charité Comprehensive Cancer Center , Medical University of Berlin, Berlin, Germany; ^23^ The Ohio State University Comprehensive Cancer Center – James Cancer Hospital and Solove Research Institute, Columbus, OH; ^24^ Biodonostia HRI, Osasun Ikerketa Insitutua, Insituto de Investigacion Sanitaria, San Sebastián, Gipuzkoa, Spain; ^25^ Department for Gynecologic Oncology and Institute for Cancer Genetics and Informatics, Oslo University Hospital, Oslo, Norway; ^26^ Department of Medical Oncology, The Christie National Health Service Foundation Trust, and University of Manchester, Manchester, United Kingdom; ^27^ Centre Léon Bérard, Netsarc Network, Université Claude Bernard Lyon 1, Lyon, France; ^28^ Belgium and Luxemburg Gynaecological Oncology Group, University Hospitals Leuven, Leuven, Belgium

## Abstract

**PURPOSE:**

Low-grade serous ovarian carcinomas (LGSOCs) have historically low chemotherapy responses. Alterations affecting the MAPK pathway, most commonly KRAS/BRAF, are present in 30%-60% of LGSOCs. The purpose of this study was to evaluate binimetinib, a potent MEK1/2 inhibitor with demonstrated activity across multiple cancers, in LGSOC.

**METHODS:**

This was a 2:1 randomized study of binimetinib (45 mg twice daily) versus physician’s choice chemotherapy (PCC). Eligible patients had recurrent measurable LGSOC after ≥ 1 prior platinum-based chemotherapy but ≤ 3 prior chemotherapy lines. The primary end point was progression-free survival (PFS) by blinded independent central review (BICR); additional assessments included overall survival (OS), overall response rate (ORR), duration of response (DOR), clinical-benefit rate, biomarkers, and safety.

**RESULTS:**

A total of 303 patients were randomly assigned to an arm of the study at the time of interim analysis (January 20, 2016). Median PFS by BICR was 9.1 months (95% CI, 7.3 to 11.3) for binimetinib and 10.6 months (95% CI, 9.2 to 14.5) for PCC (hazard ratio,1.21; 95%CI, 0.79 to 1.86), resulting in early study closure according to a prespecified futility boundary after 341 patients had enrolled. Secondary efficacy end points were similar in the two groups: ORR 16% (complete response [CR]/partial responses[PRs], 32) versus 13% (CR/PRs, 13); median DOR, 8.1 months (range, 0.03 to ≥ 12.0 months) versus 6.7 months (0.03 to ≥ 9.7 months); and median OS, 25.3 versus 20.8 months for binimetinib and PCC, respectively. Safety results were consistent with the known safety profile of binimetinib; the most common grade ≥ 3 event was increased blood creatine kinase level (26%). Post hoc analysis suggests a possible association between *KRAS* mutation and response to binimetinib. Results from an updated analysis (n = 341; January 2019) were consistent.

**CONCLUSION:**

Although the MEK Inhibitor in Low-Grade Serous Ovarian Cancer Study did not meet its primary end point, binimetinib showed activity in LGSOC across the efficacy end points evaluated. A higher response to chemotherapy than expected was observed and *KRAS* mutation might predict response to binimetinib.

## INTRODUCTION

Serous carcinoma accounts for approximately 70%-80% of epithelial ovarian, tubal, and peritoneal cancers.^1^ Low-grade serous ovarian carcinoma (LGSOC) is a unique tumor that is distinguished from high-grade serous ovarian cancer not only by immunohistochemical profile but also by molecular characteristics, epidemiologic features and clinical behavior.^2^

**CONTEXT**

**Key Objective**
The objective of the MEK Inhibitor in Low-Grade Serous Ovarian Cancer (MILO)/ENGOT-ov11 study was to evaluate the MEK1/2 inhibitor binimetinib in patients with low-grade serous ovarian carcinomas (LGSOCs).
**Knowledge Generated**
This study did not meet its primary end point; however, binimetinib showed activity in LGSOC across the efficacy end points evaluated. Chemotherapy responses were higher than predicted. The safety results observed in this study are generally consistent with the known safety profile of binimetinib and with MEK inhibitor class effects.
**Relevance**
Currently, treatment options are limited for patients with LGSOC, and few offer objective decreases in disease burden or tumor-progression delays. Although this trial did not meet its primary end point, binimetinib did display a clinically meaningful progression-free survival and overall response rate and, therefore, should be considered a viable treatment option in this setting. Forthcoming biomarker analysis may ultimately identify a subset of patients who selectively benefit from binimetinib.

Aberrant signaling through the RAS/RAF/MEK/ERK pathway is a characteristic feature of many cancers, including LGSOC, with, 5%-16% and 16%-47% of LGSOCs having alterations in BRAF and RAS, respectively.^3-7^

Binimetinib is an oral, potent, selective, allosteric, small-molecule inhibitor of MEK1/2 and is approved in multiple countries in combination with encorafenib for the treatment of patients with unresectable or metastatic *BRAF* V600E or V600K mutation-positive melanoma.^8,9^ Inhibiting both basal and induced levels of ERK phosphorylation in numerous *BRAF*-mutated cancer cell lines (half maximal inhibitory concentration [IC_50_] values as low as 5 nM), binimetinib has nanomolar activity against purified MEK enzyme (IC_50_, 12 nM). Binimetinib has also demonstrated a decrease in pERK when tested in multiple cell lines, regardless of their mutational status and in vitro sensitivity.^10^ A prior single-arm, phase II study of the MEK inhibitor selumetinib showed promising activity in recurrent LGSOC.^11^

This phase III study was designed to evaluate the efficacy and safety of binimetinib in recurrent or persistent LGSOC. Patients were not selected on the basis of molecular profile; however, archival tumor tissue was collected at the time of enrollment for retrospective mutational analysis. Blinded independent central radiology review (BICR) was used to control for potential investigator variance in assessing response.

## PATIENTS AND METHODS

### Patients

Patients were > 18 years of age with a diagnosis of LGSOC, fallopian tube or primary peritoneum, confirmed histologically and verified by central pathology review. Archival tissue was also collected for biomarker testing using the FoundationOne Panel (Foundation Medicine, Cambridge, MA). Eligible patients had measurable recurrent or persistent disease (as defined by RECIST V1.1, per BICR) that had progressed (defined as radiologic and/or clinical progression; an increase in CA-125 alone was not sufficient) on or after last therapy, and was not amenable to potentially curative intent surgery, as determined by the investigator. Patients were required to have received ≥ 1 prior platinum-based chemotherapy regimen but ≤ 3 prior chemotherapy regimens in total, with no limit to the number of lines of prior hormonal therapy. Patients had an Eastern Cooperative Oncology Group performance status of 0 or 1. Patients were excluded if they had previous treatment with an MEK or BRAF inhibitor. Additional details regarding inclusion and exclusion criteria are provided in the Data Supplement.

The study was approved by the institutional review board for each site. All clinical work was conducted in compliance with current Good Clinical Practices as referenced in the International Conference on Harmonization of Technical Requirements for Registration of Pharmaceuticals for Human Use. All patients enrolled in the study provided written, informed consent prior to their participation.

### Study Design and Treatments

The MEK Inhibitor in Low-Grade Serous Ovarian Cancer (MILO)/ARRAY-162-311/ENGOT-ov11 study was a multinational, randomized, two-arm, open-label, phase III study conducted at 102 sites in 20 countries (ClinicalTrials.gov identifier: NCT01849874; Appendix Table A1, online only). MILO was conducted in collaboration with European Network of Gynecologic Oncological Trial groups (ENGOT) according to the ENGOT Model C.^12^ Patients were stratified by their last platinum-free interval (≤ *v* > 182 days) and number of prior systemic regimens (1 to 2 *v* 3) and then randomly assigned 2:1 to receive binimetinib or physician’s choice chemotherapy (PCC; pegylated liposomal doxorubicin [PLD], paclitaxel, or topotecan). Patients randomly assigned to binimetinib received 45 mg orally twice daily with water irrespective of food, continuously, starting on day 1. Patients randomly assigned to PCC received one of the following: PLD (40 mg/m^2^ intravenously [IV] on day 1 of every 28-day cycle), paclitaxel (80 mg/m^2^ IV on days 1, 8, and 15 of every 28-day cycle), or topotecan (1.25 mg/m^2^ IV on days 1-5 of every 21-day cycle). Treatment continued until one of the following: locally determined progressive disease (PD) unacceptable toxicity, or inability to continue on protocol-directed therapy (additional information is provided in the Data Supplement). Patients randomly assigned to PCC who developed PD (by local and BICR assessment) were allowed to crossover to treatment with binimetinib provided they met the crossover eligibility requirements (Data Supplement).

### Assessments

The primary end point was BICR progression-free survival (PFS). Secondary end points included overall survival (OS), overall response rate (ORR; RECIST v1.1), duration of response (DOR), disease control rate (best response of complete response [CR] or partial response [PR], or stable disease [SD] documented ≥ week 24) and safety.

Tumors were assessed every 8 weeks for the first 72 weeks, then every 12 weeks until PD per BICR, irrespective of the days of study- drug administration. Safety was evaluated by ongoing monitoring, including ophthalmic examinations, dermatologic examinations, electrocardiograms, and cardiac scans of ejection fraction.

### Statistical Methods

For efficacy, all randomly assigned patients were included in the analyses. For safety, all patients who received binimetinib or PCC were included. PFS was defined as the date of randomization to the date of first documented BICR PD or death due to any cause, whichever occurred first. If a patient had not experienced an event at the time of the analysis cutoff or at the start of any new therapy, PFS was censored at the date of last adequate tumor assessment. PFS and OS were summarized by treatment arm using the Kaplan-Meier method with 95% CIs for medians. The primary end point was compared between treatment arms using a stratified log-rank test, and a hazard ratio [HR] from the stratified Cox model was used to summarize the treatment effect estimate.

ORR was assessed and compared between arms using the Fisher exact test. Median DOR with 95% CIs was provided, with minimum, maximum, and the number still in response (censored) at the time of data cutoff.

A total of 195 events (PD or death) provided 90% power for testing the null hypothesis of no difference in PFS distribution functions between the two treatment arms assuming a true HR of 0.60 using a stratified log-rank test, a 1-tailed α of 0.025, and a 2:1 binimetinib arm to control arm randomization ratio. The HR required to achieve the final critical value was approximately 0.74. Historical evidence suggests that the median PFS in recurrent LGSOC is approximately 7 months.^6,7^ For exponential PFS, a HR of 0.60 translates to a median PFS of approximately 11.7 months in the binimetinib arm. A total of approximately 360 patients were planned. An interim analysis for early stopping for futility was planned at 40% information fraction (ie, n = 78 total progression events per BICR or deaths). The futility boundary was from the unified family of group sequential test designs with parameter *P* = 0.5.^13^ At 40% information fraction, this corresponds to an approximate boundary of 0.90 on the HR scale. A data cutoff date was set by the sponsor in advance of the occurrence of the 78th event. FoundationOne Panel genes that were prevalent in at least 5% of sequenced patients were tested for association with binary response (CR or PR *v* SD or PD) using two-sided Fisher exact tests.

## RESULTS

### Patient Characteristics and Drug Exposure

**FIG 1.**
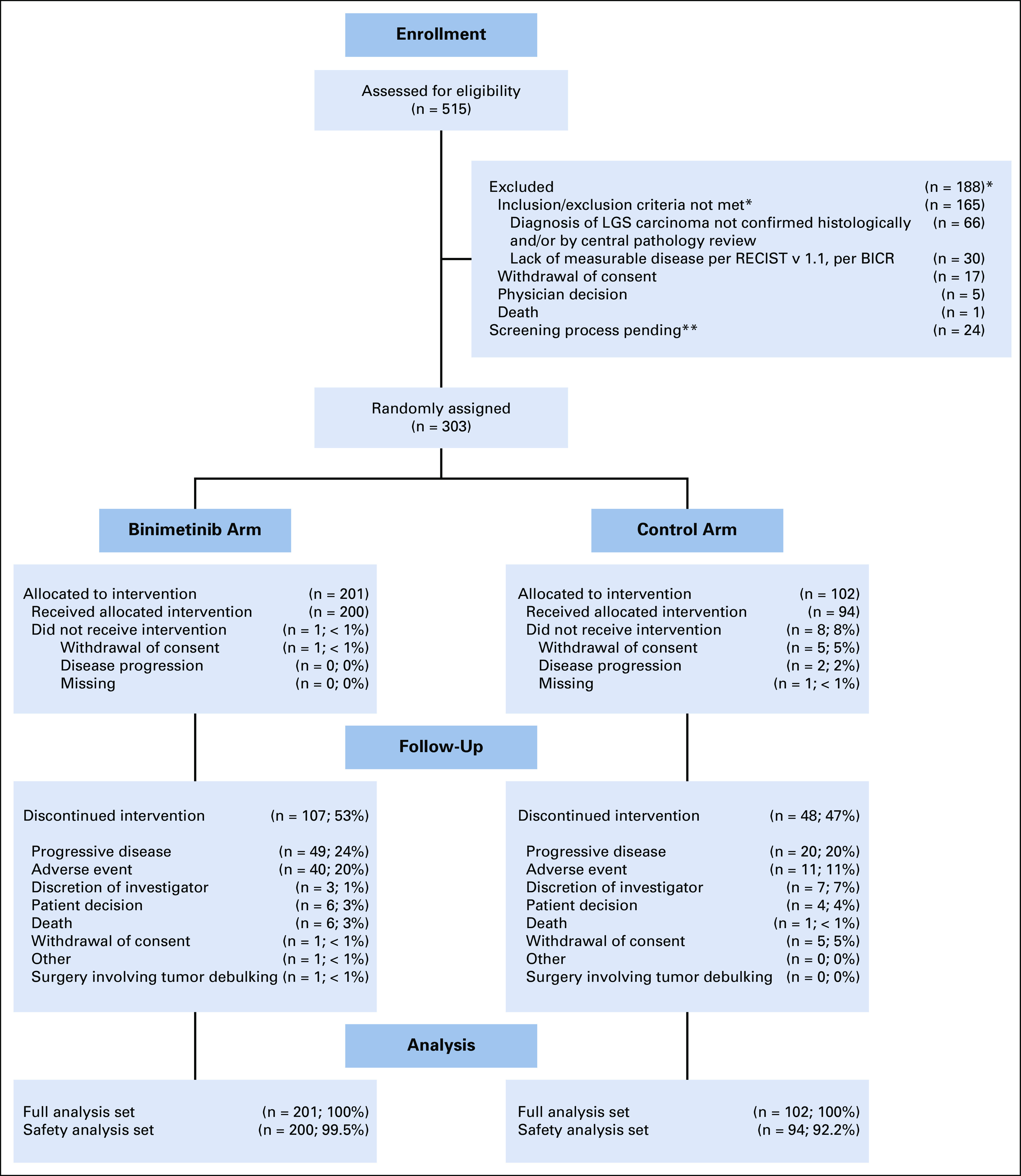
CONSORT diagram (data cutoff date: January 20, 2016). BICR, blinded independent central review; LGS, low-grade serous. (*) Patients may be counted as not meeting > 1 criterion; most common reasons provided. (**) These patients had signed ICF prior to the data cutoff date but the outcome of their screening process was still pending as of the cutoff date.

Patients were enrolled from June 28, 2013, to April 1, 2016. Per recommendation of the data monitoring committee, enrollment was discontinued after the planned interim analysis showed the HR for PFS crossed the predefined futility boundary. The interim analysis was conducted with 303 patients and then, at the time of the decision to discontinue enrollment for the study, 341 patients. Results presented here include an assessment of end points during the randomized period, up to the data cutoff date for the interim analysis of January 20, 2016, for a total of 303 patients (n = 201 patients receiving binimetinib; n = 102 receiving PCC) in the full analysis set and 294 patients (n = 200 receiving binimetinib; n = 94 receiving PCC) in the safety population (Fig 1). At the time of data cutoff, (January 20, 2016), 107 patients (53%) and 48 patients (47%) had discontinued treatment of binimetinib and PCC, respectively. The most common reasons for discontinuing initial treatment were disease progression (binimetinib, 24%; PCC, 20%) and adverse events (binimetinib, 20%; PCC, 11%; Fig 1). Patient baseline demographics and disease characteristics were generally well balanced between the two groups (Table 1).

**TABLE 1.**
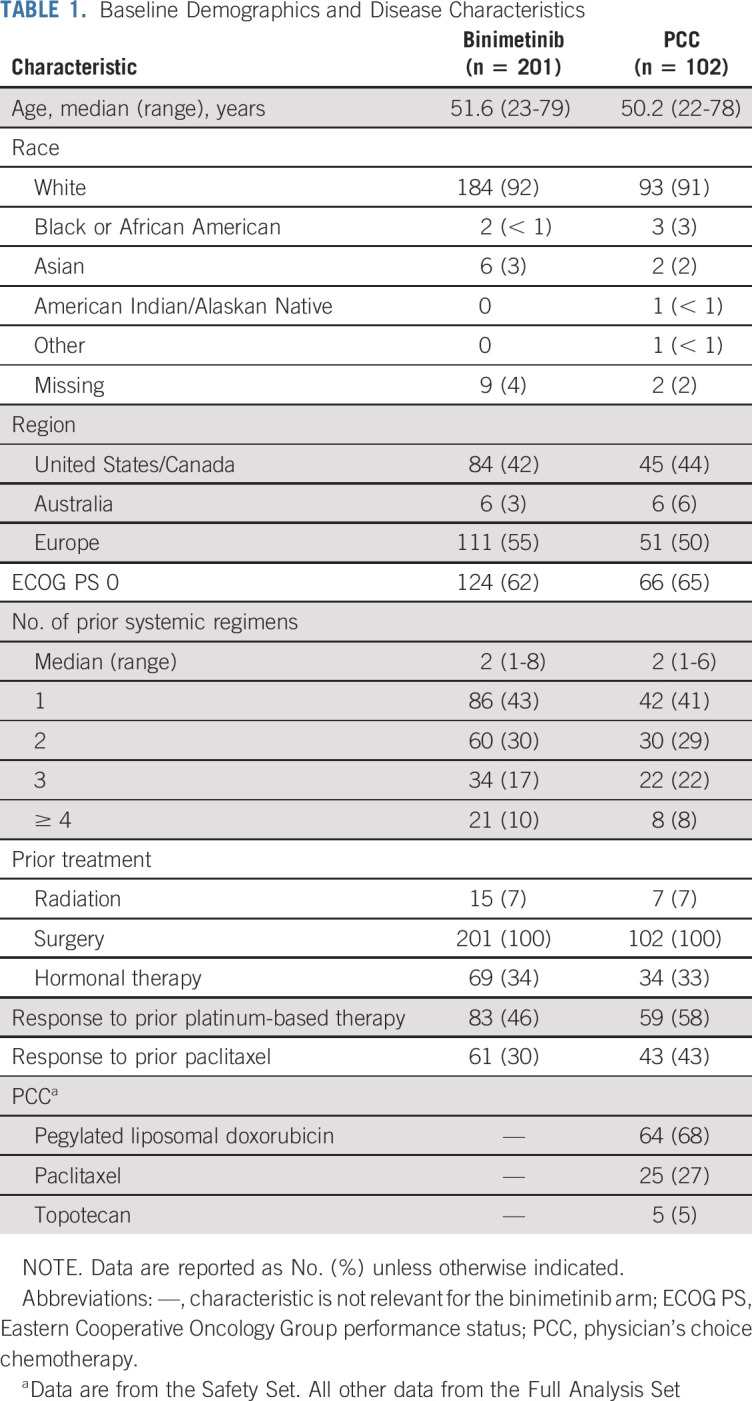
Baseline Demographics and Disease Characteristics

The median duration of exposure to binimetinib was 4.1 months (range, 0-24 months) and the median relative dose intensity was 67.6% (range, 6%-100%). The median duration of exposure to any of the PCC was 4.1 months (range, 0-18 months). Patients in the PCC group received PLD (n = 64 patients; 68%); paclitaxel (n = 25 patients; 27%), or topotecan (n = 5 patients; 5%). The median (range) relative dose intensity was 71.3% (40%-100%) for topotecan, 95.9% (0%-116%) for PLD, and 89.4% (33%-102%) for paclitaxel.

### Efficacy

**FIG 2.**
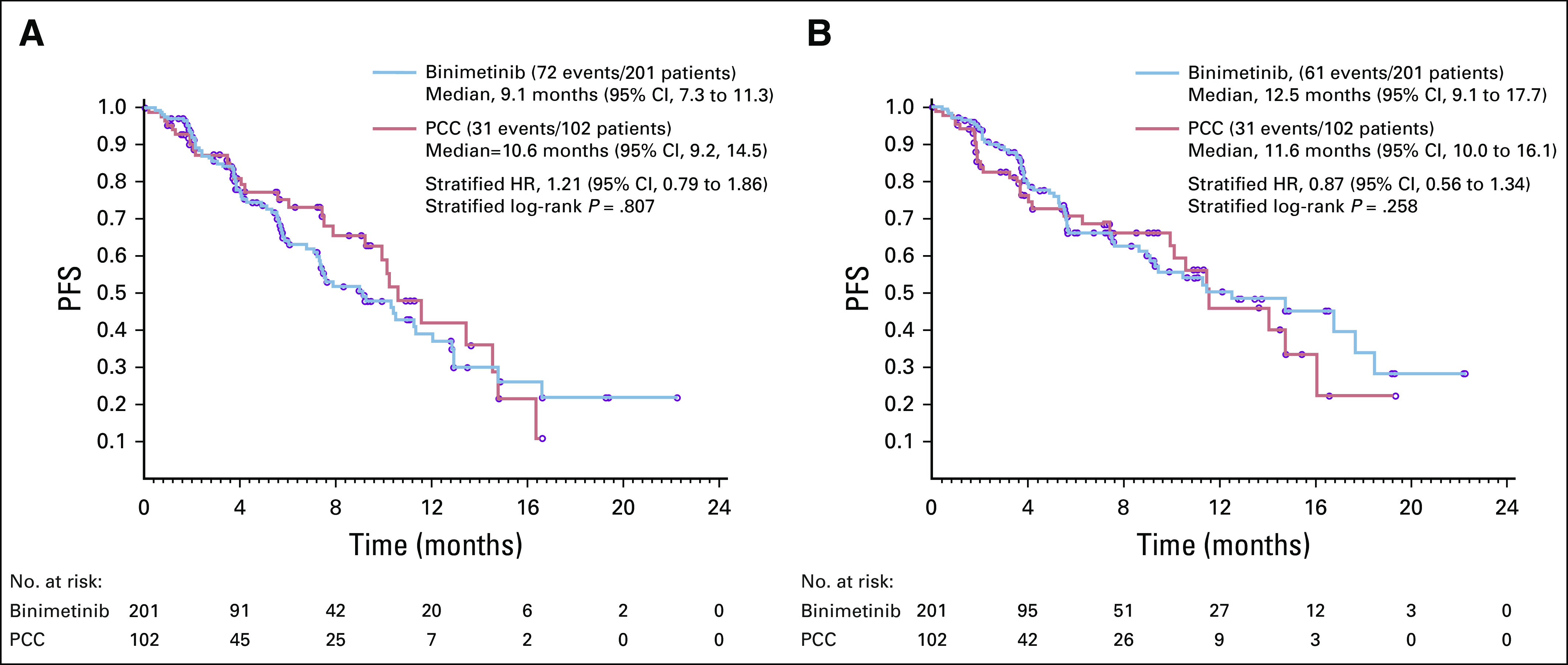
Kaplan-Meier plot of progression-free survival per (A) blinded independent central review and (B) local assessment. HR, hazard ratio; PCC, physician’s choice chemotherapy; PFS, progression free survival.

The primary end point of PFS by BICR is shown in Figure 2. The median PFS was 9.1 months (95% CI, 7.3 to 11.3) in the binimetinib group and 10.6 months (95% CI, 9.2 to 14.5) in the PCC group. The HR from the stratified Cox model was 1.21 (95% CI, 0.79 to 1.86). Based on a point-estimate futility boundary of HR > 0.84 for the 103 events observed in the interim analysis, the futility boundary was crossed, indicating a low probability of reaching statistical significance in favor of binimetinib with continued follow-up. In the local investigator assessment, patients in the binimetinib arm had a median PFS of 12.5 months (95% CI, 9.1 to 17.7) compared with 11.6 months (95% CI, 10.0 to 16.1) in the PCC group. The stratified HR was 0.87 (95% CI, 0.56 to 1.34).

The OS results are depicted in the Data Supplement. They were similar between groups, with 164 patients (82%) in the binimetinib group alive at the time of data cutoff for interim analysis compared with 82 patients (80%) in the PCC group. The median OS was 25.33 months (95% CI, 18.46 to not reached [NR]) in the binimetinib group and 20.83 months (95% CI, 17.45 to NR) in the PCC group. The HR from the stratified Cox model was 0.85 (95% CI, 0.49 to 1.48).

The response analysis is shown in Table 2. The ORR by BICR was 16% in the binimetinib group and 13% in the PCC group. The median DOR in the binimetinib group was 8.05 months (95% CI, 5.55 to NR) compared with 6.67 months (95% CI, 3.71 to NR) in the PCC group; 23 patients in the binimetinib group and 8 patients in the PCC group had responses ongoing at the data cutoff date. For the response assessment by local investigator, the ORR was 18% in the binimetinib group and 13% in the PCC group. Median DOR was 15.84 months (95% CI, 10.41 to NR) in the binimetinib group and 9.89 months (95% CI, 6.41 to 9.89) in the PCC group. A waterfall plot displaying percent change in sum of longest diameters per BICR is displayed in the Data Supplement.

**TABLE 2.**
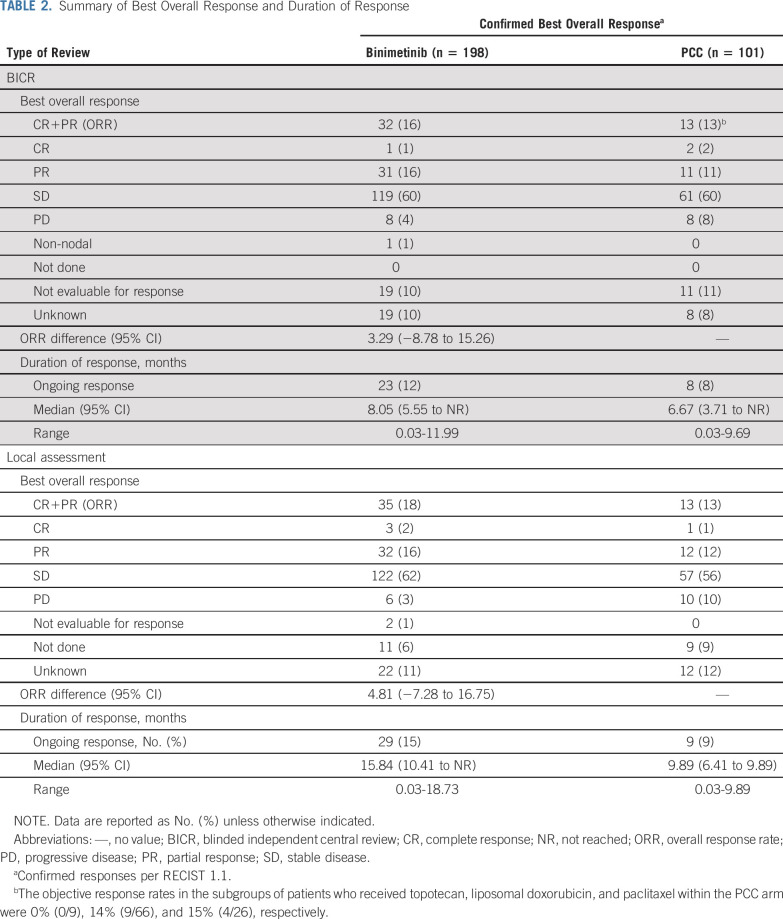
Summary of Best Overall Response and Duration of Response

At the time enrollment to the study ended in April 2016, patients being treated with binimetinib or PCC were notified of the interim results, but if desired, they were allowed to continue receiving treatment until treatment discontinuation criteria were met. Crossover was stopped at that time. An updated analysis was conducted when the remaining data were collected after the discontinuation of enrollment, with a data cutoff of January 2019 (n = 341). In this analysis, median PFS by BICR was 10.4 months (95% CI, 7.5 to 12.9) in the binimetinib group and 11.5 months (95% CI, 9.9 to 14.8) in the PCC group (HR, 1.15;95% CI, 0.76 to 1.74; Data Supplement). Median OS was 34.6 months (95% CI, 28.0 to NR) and 34.2 months (95% CI, 21.6 to NR) for the binimetinib and PCC groups, respectively (HR, 0.93; 95% CI, 0.65 to 1.33; Data Supplement). Updated ORR by local investigator assessment was 24% in both groups (Data Supplement). It is important to note the median OS estimates in both arms increased at the follow-up analysis, possibly as a result of the instability of the median estimates at the time of the initial analysis, when the potential follow-up was substantially (3 years) shorter.

**FIG 3.**
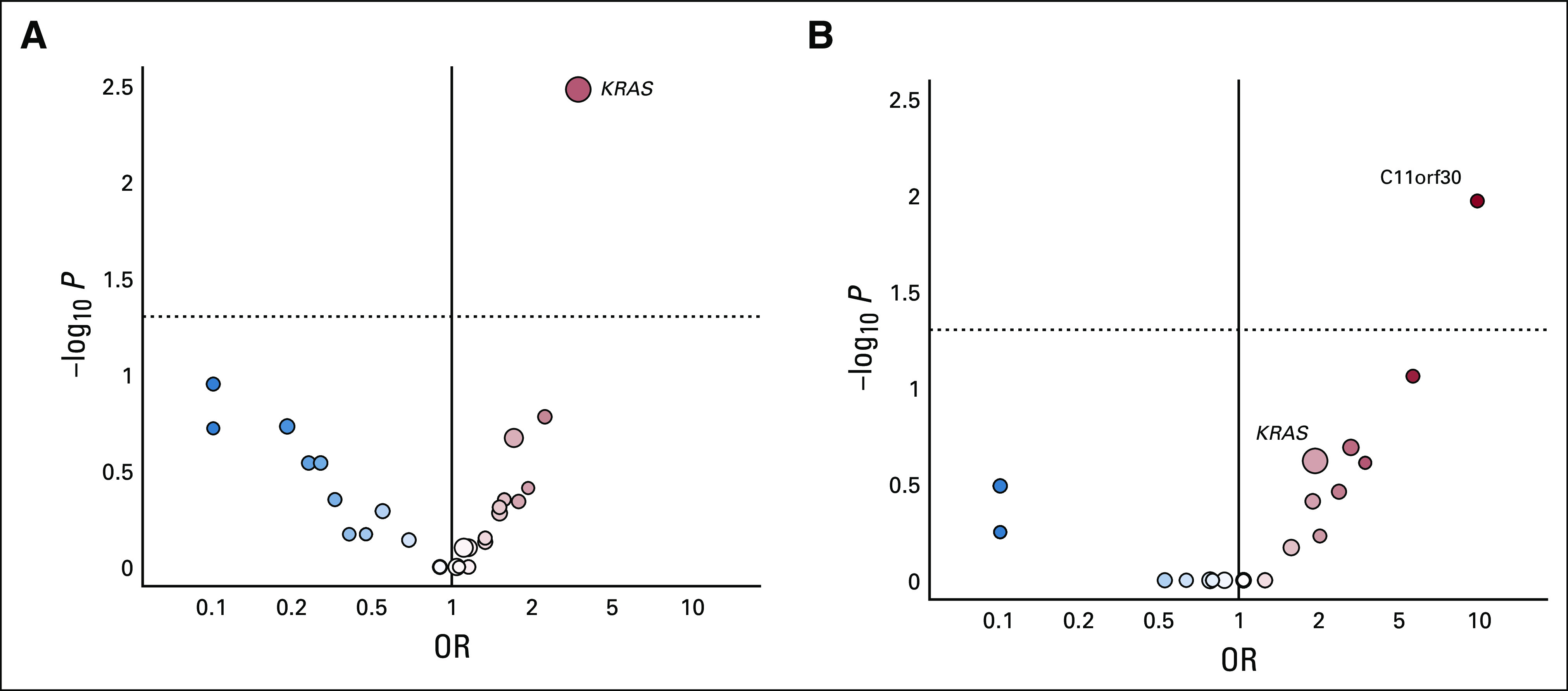
(A) Binimetinib treatment group: univariate analysis of molecular alterations and response to therapy. (B) Physician’s choice chemotherapy group: univariate analysis of molecular alterations and response to therapy. OR, odds ratio.

Molecular testing was performed on all consenting patients with adequate archival tissue. At the time of the January 2019 data cutoff, 215 patients had tumor tests available. There were 47 mutations detected in at least 5% of patients, most commonly *KRAS*, which was found in 33% of patients. The frequency of *KRAS* mutation was evenly distributed between the two groups and was found in 46 patients (32%) treated with binimetinib and 24 patients (34%) treated with PCC. Unbiased univariate analyses evaluating best ORR to therapy as a binary response showed *KRAS* mutation was significantly associated with response to treatment with binimetinib (odds ratio [OR], 3.4; 95% CI, 1.53 to 7.66; unadjusted *P* = .003; Fig 3A) but not PCC (OR, 2.13; 95% CI, 0.67 to 6.81; *P* = .2; Fig 3B). *KRAS* mutation was also associated with prolonged PFS in patients treated with binimetinib (median PFS: *KRAS* mutant: 17.7 months [95% CI, 12 to NR]; KRAS wild-type (WT): 10.8 months [95% CI, 5.5 to 16.7]; *P* = .006), but not PCC (median PFS: *KRAS* mutant: 14.6 months [95% CI, 9.4 to NA]; *KRAS* WT: 11.5 months [95% CI, 5.7 to 26.6]; *P* = .502).Among those patients treated with binimetinib for whom updated local RECIST 1.1 response data and molecular data were available (n = 133), *KRAS* mutation status was significantly associated with local best response (*P* = .004); 44% of patients with *KRAS* mutation versus 19% of patients with *KRAS* WT had CR or PR (Table 3). Mutations identified by Foundation Medicine FoundationOne Panel in ≥ 1 tumor sample are listed in the Data Supplement.

**TABLE 3.**
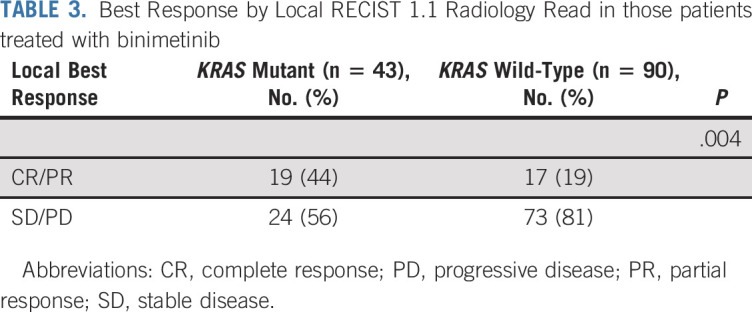
Best Response by Local RECIST 1.1 Radiology Read in those patients treated with binimetinib

### Safety

Grade ≥ 3 adverse events were reported in 76% and 44% of patients for binimetinib and PCC, respectively (Table 4). Adverse events that led to permanent discontinuation of study drug were reported by 62 patients (31%) for binimetinib and 16 patients (17%) in the PCC group. Adverse events leading to binimetinib discontinuation in ≥ 5 patients were decreased ejection fraction (n = 8 patients; 4%), vomiting (n = 6 patients; 3%), intestinal obstruction and retinal vein occlusion (n = 5 patients; 2% each). The adverse event leading to discontinuation of PCC in ≥ 5 patients was palmar-plantar erythrodysesthesia syndrome (n = 5 patients; 5%). A total of six patients (3%) in the binimetinib group experienced a retinal vein occlusion event, all of which resulted in treatment discontinuation. All events were considered resolved or resolving, two with sequelae. No permanent blindness or permanent loss of vision was observed.

**TABLE 4.**
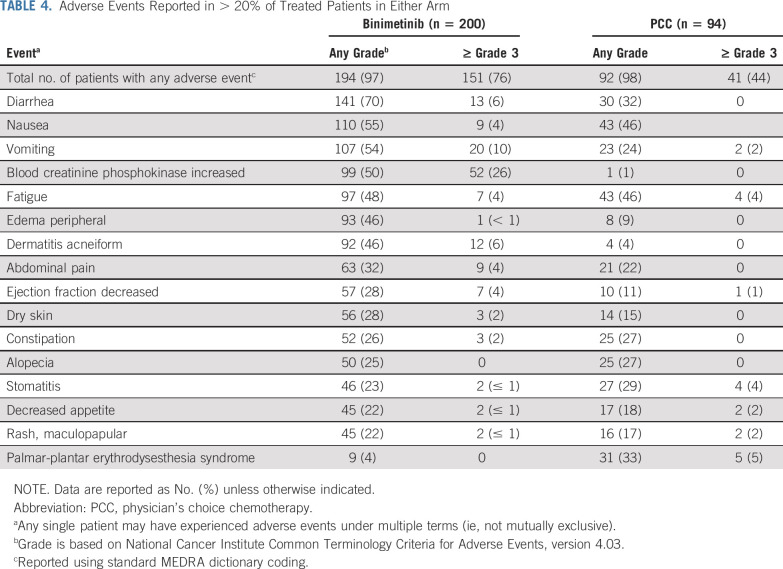
Adverse Events Reported in > 20% of Treated Patients in Either Arm

## DISCUSSION

Binimetinib did not demonstrate a significant difference in the primary end point of PFS versus PCC in patients with recurrent or persistent LGSOC. In addition, the proportion of patients achieving an objective response and the median DOR appeared similar between arms. Of note, the responses to chemotherapy in this study were greater than anticipated on the basis of previously reported, single-institution retrospective case series.

Although the MILO/ENGOT-ov11 trial did not meet its primary end point, binimetinib did display a clinically meaningful PFS and ORR and, therefore, should be considered a viable treatment option in this setting. The median OS for patients with advanced LGSOC approaches 10 years, with patients often experiencing significant morbidity from their disease during that time.^2^ Currently, treatment options are limited for patients with this disease and few offer objective decreases in disease burden or delays in tumor progression. Recent results from another phase II/III trial in 260 patients with recurrent LGSOC showed trametinib was associated with significantly improved PFS (median, 13.0 *v* 7.2 months; HR, 0.48; 95% CI, 0.36 to 0.64; *P* < .0001) and ORR (trametinib: 26.2% *v* control:6.2%; OR, 5.4; 95% CI, 2.39 to 12.21; *P* < .0001) compared with physician’s choice standard of care, also indicating the potential of MEK inhibition in this patient population.^14^ Of note, the control arm in that study did not appear to perform as well as in the current study, possibly because of differences in inclusion criteria. The trametinib study allowed for an unlimited number of prior chemotherapies, whereas the binimetinib study was limited to patients who had received a maximum of three prior lines of chemotherapy. Differences in study design and inclusion criteria likely selected for a more chemotherapy-resistant population in the trametinib study, explaining the similar activity of MEK inhibitors between the two studies (response rate of 24% on updated analysis of binimetinib study; 26.2% in the trametinib study) but difference in activity within the control arms. Safety results from this study show that patients treated with binimetinib had higher rates of nonserious and serious adverse events overall, as well as grade ≥ 3 adverse events compared with the PCC group, and there were more frequent dose reductions, dose interruptions, and permanent discontinuations due to adverse events experienced by patients in the binimetinib group, resulting in a lower relative dose intensity for the binimetinib group compared with any of the drugs in the PCC group. The majority of adverse events assessed as related to binimetinib were reversible with or without drug interruption. The safety profile observed in this study is consistent with the known binimetinib profile and consistent with those for the class of MEK inhibitors.^15^

There are several limitations of the study. First, the lack of suitable, validated biomarkers led to a design with an unselected patient population. Post hoc analysis suggests a possible association between *KRAS* mutation and response to binimetinib. Additional exploration is warranted to determine if patients with *KRAS* mutation may derive greater benefit from binimetinib. Although KRAS has been an elusive target across multiple cancer types, prior early-phase studies have found promising response rates to MEK inhibitors and MEK inhibitor combinations in those patients with *KRAS*-mutant LGSOC.^16-18^ This has led to considerable interest in the use of mutation status when weighing the expected adverse effects versus benefits of MEK inhibitor therapy. Adverse events in the binimetinib group led to study discontinuations and a low dose intensity. The safety events noted in this study were resolved with conservative supportive care and could potentially be mitigated in future protocols with more proactive management.

In conclusion, although this study did not meet its primary end point, binimetinib showed activity in LGSOC across the efficacy end points evaluated. Chemotherapy responses were greater than predicted. The safety results observed in this study are generally consistent with the known safety profile of binimetinib and with MEK inhibitor class effects. Forthcoming biomarker analysis may ultimately identify a subset of patients who selectively benefit from binimetinib, and additional clinical evaluation is warranted.

## Figures and Tables

**TABLE A1.**
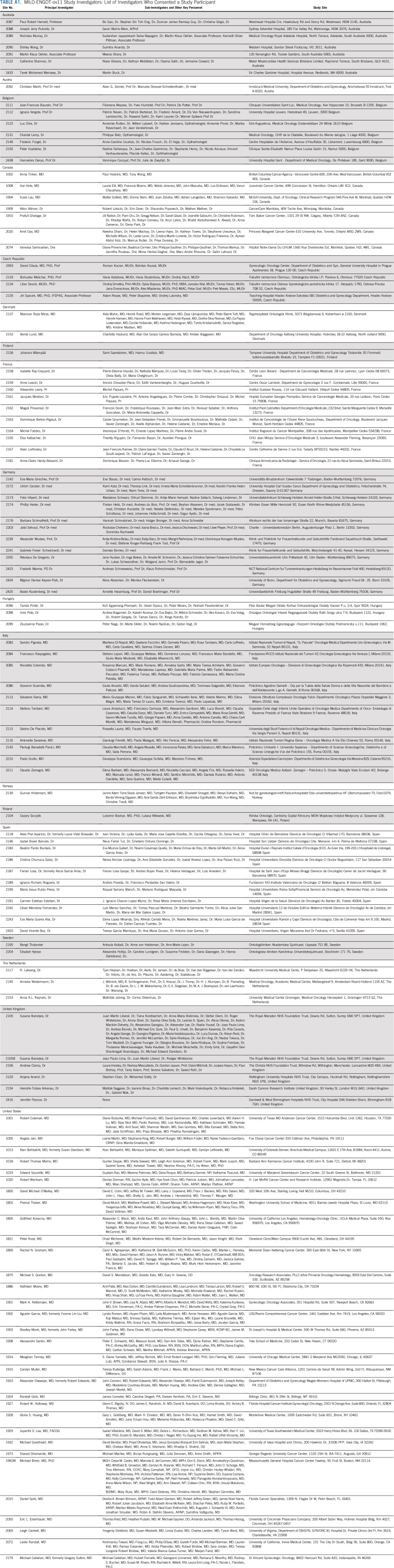
MILO-ENGOT-ov11 Study Investigators: List of Investigators Who Consented a Study Participant
